# Topical Pregabalin in Burning Mouth Syndrome: A Real-World Study of Peripheral Neuromodulation

**DOI:** 10.3390/medicina62071299

**Published:** 2026-07-05

**Authors:** Federica Canfora, Antonietta Argiuolo, Simona Salerno, Claudia Castellucci, Roberta Evangelista, Salvatore Ferrara, Rosa Valletta, Alfredo De Rosa, Lucia Memè, Michele Davide Mignogna, Daniela Adamo

**Affiliations:** 1 Department of Neurosciences, Reproductive Sciences and Odontostomatology, University of Naples “Federico II”, 80131 Naples, Italy; dottsimonasalerno@gmail.com (S.S.); claudiacastellucci@outlook.it (C.C.); roberta.evangelista7@gmail.com (R.E.); ferraradrsalvatore@gmail.com (S.F.); valletta@unina.it (R.V.); mignogna@unina.it (M.D.M.); d.adamo@unilink.it (D.A.); 2 Departmental Program of Clinical Psychopathology, Federico II University Hospital, 80131 Naples, Italy; tonia.argiuolo@outlook.it; 3 Department of Life Science, Health and Health Professions, Link Campus University, 00165 Rome, Italy; a.derosa@unilink.it (A.D.R.); l.meme@unilink.it (L.M.)

**Keywords:** Burning Mouth Syndrome, pregabalin, topical therapy, neuropathic pain, chronic oral pain, real-world study, Visual Analog Scale, anxiety, depression, sleep disturbance, neuromodulatory treatment

## Abstract

*Background and Objectives:* Burning Mouth Syndrome (BMS) remains a challenging condition to manage, as current treatments show variable efficacy and are often limited by tolerability, particularly in older and medically complex patients. This has prompted interest in topical neuromodulatory strategies targeting peripheral mechanisms while minimizing systemic exposure, with topical pregabalin emerging as a potential option. *Materials and Methods:* In this prospective longitudinal real-world study, 100 patients with BMS treated at a tertiary referral center received topical pregabalin as an off-label intraoral swish-and-spit solution. Patients were assessed at baseline and after 3 months using validated measures of pain intensity (VAS), qualitative pain perception (SF-MPQ), anxiety (HAM-A), depression (HAM-D), sleep quality (PSQI, ESS), and global clinical severity (CGI-S). *Results:* After 3 months, median VAS decreased from 8 (IQR 7–9) to 5 (IQR 4–6) and SF-MPQ from 10 (IQR 7–17) to 6.5 (IQR 4–10) (both *p* < 0.001), with concurrent improvements in anxiety, depressive symptoms, and clinical severity. Overall, 46% of patients achieved ≥30% pain reduction, while 73% and 16% reached ≥20% and ≥50% reductions, respectively. Higher baseline pain predicted greater improvement. No serious adverse events were reported. *Conclusions:* These findings suggest that topical pregabalin may represent a safe and potentially effective option for BMS, although controlled studies are required to confirm its efficacy.

## 1. Introduction

Burning Mouth Syndrome (BMS) is a chronic intraoral pain disorder characterized by persistent burning or dysesthetic sensations of the oral mucosa in the absence of clinically evident lesions or identifiable local or systemic causes [1]. The condition affects approximately 1–3% of the general population, with a marked predominance in peri- and post-menopausal women, in whom prevalence rates may be substantially higher in some cohorts [2]. Recent systematic reviews and meta-analyses confirm that BMS is more frequent in older females and represents one of the most common chronic oral pain disorders encountered in clinical practice [3,4,5].

Available epidemiological data suggests that the prevalence and clinical characterization of BMS may vary across geographical regions, although evidence remains unevenly distributed [6]. A recent worldwide systematic review reported differences in prevalence estimates across continents, with available data mainly from Europe, North America, and selected Asian populations [7,8]. Population-based and clinical studies from Asia, including Taiwan, China, and Korea, as well as emerging data from Africa and Eastern Europe, confirm the global relevance of BMS but also highlight heterogeneity in diagnostic criteria, study design, and healthcare settings [3,7]. Therefore, further geographically diverse and methodologically harmonized studies are needed to better define regional differences in BMS prevalence, clinical presentation, and management [9].

In addition to the characteristic burning sensation, patients frequently report xerostomia, dysgeusia, globus pharyngeus, and foreign-body sensation [10]. Beyond the sensory component, BMS is strongly associated with sleep disturbances, anxiety, and depressive symptoms, highlighting the multidimensional nature of the disorder and its significant impact on quality of life [4,11].

Despite its relatively high prevalence, the pathophysiology of BMS is still incompletely understood, and its management remains largely empirical. Increasing evidence supports the concept that BMS represents a neuropathic pain condition involving both peripheral and central mechanisms [12,13]. Histological studies have demonstrated reduced intraepithelial nerve fiber density and alterations in small-fiber function within the oral mucosa, while neurophysiological and functional imaging findings suggest abnormalities in trigeminal sensory processing [14] and central pain modulation pathways. These observations support the current view of BMS as a heterogeneous disorder in which peripheral sensitization, central disinhibition, and psychological factors may coexist, with variable contributions across individual patients [15].

Because of this complex pathophysiology, pharmacological treatment of BMS remains challenging and often unsatisfactory. Systemic therapies commonly used in neuropathic pain, including antidepressants, benzodiazepines, and anticonvulsants, have shown variable responses and are frequently limited by adverse effects, particularly in older patients with multiple comorbidities [16,17]. These limitations have led to increasing interest in therapeutic strategies aimed at targeting peripheral nociceptive mechanisms while minimizing systemic exposure.

Topical clonazepam, administered with a swish-and-spit technique, represents the most extensively studied local treatment and has demonstrated benefit in several clinical trials and observational studies, although response rates remain heterogeneous and systemic adverse effects may still occur due to partial mucosal absorption [18]. More recently, other topical neuromodulator agents have been investigated, including amitriptyline and gabapentin.

Recent studies evaluating topical amitriptyline have reported improvement in burning symptoms and quality-of-life measures. Similarly, preliminary real-world data suggest that topical gabapentin may provide clinically meaningful pain reduction with acceptable tolerability, supporting the role of peripheral neuromodulation in BMS [19], and further reinforcing the hypothesis that local modulation of oral sensory nerve endings may represent an effective therapeutic strategy in selected patients [20,21].

Gabapentinoids, such as gabapentin and pregabalin (PGB), are widely used in neuropathic pain disorders due to their ability to bind the alpha-2-delta (α2δ) subunit of voltage-gated calcium channels, thereby reducing excitatory neurotransmitter release and attenuating peripheral and central sensitization [22]. However, systemic administration of these agents in BMS has produced variable results and may be associated with dizziness, somnolence, and cognitive impairment, which often limit long-term adherence [19].

PGB is a second-generation gabapentinoid with higher affinity for the α2δ subunit of voltage-gated calcium channels and more predictable pharmacokinetic properties than gabapentin [23]. Although systemic pregabalin has shown efficacy in several neuropathic pain conditions, including BMS, evidence regarding its topical use in BMS and other neuropathic pain disorders remains limited, and most available studies have focused on systemic administration [21,22,23]. To date, no studies have comprehensively investigated the clinical effects of topical pregabalin (tPGB) in a well-characterized cohort of patients with BMS. Considering the potential role of peripheral neuropathic mechanisms in BMS, local administration of pregabalin may represent a promising therapeutic strategy capable of providing targeted neuromodulation while reducing the risk of systemic adverse effects.

Recently, experimental pregabalin formulations for intraoral use have shown good tolerability together with a reduction in sensitivity in patients undergoing dental bleaching. In addition, in vitro studies have demonstrated acceptable cytocompatibility of these formulations on oral tissues, supporting the feasibility of topical delivery within the oral cavity [24,25]. Furthermore, topical pregabalin has shown analgesic effects in experimental models of neuropathic orofacial pain, reducing nociceptive behavior with minimal systemic exposure [26,27].

In this context, topical PGB (tPGB) represents a biologically plausible therapeutic option in clinical settings, as it may allow targeted modulation of trigeminal afferents at the intraoral level while potentially minimizing the dizziness, somnolence, and cognitive adverse effects commonly associated with systemic gabapentinoid therapy [28]. This aspect is particularly relevant in patients with BMS, who are typically older, frequently present with multiple comorbidities, and are often exposed to polypharmacotherapy.

However, despite this strong pharmacological and pathophysiological rationale, clinical evidence on tPGB in BMS remains limited. Therefore, this prospective longitudinal real-world study aimed to evaluate the clinical effects of tPGB in a large cohort of patients with BMS treated at a tertiary referral center. Changes in pain intensity, qualitative pain perception, psychological symptoms, sleep quality, and global clinical severity were assessed over a 3-month follow-up period to better characterize its therapeutic impact and potential role as a peripheral neuromodulatory strategy.

## 2. Materials and Methods

### 2.1. Study Design and Setting

This was a prospective, longitudinal, real-world, single-arm observational study conducted at the Orofacial Pain Unit of the University of Naples Federico II, a tertiary referral center for the diagnosis and management of chronic oral pain disorders.

Patients were consecutively enrolled between February and March 2025 and followed for 3 months, with final assessments completed in June 2025. The study aimed to evaluate the clinical effects of topical pregabalin under routine clinical practice conditions, without modification of the standard therapeutic approach adopted in our center.

The study was conducted in accordance with the ethical standards of the institutional research committee and the Declaration of Helsinki and its later amendments. The protocol was approved by the Ethics Committee of the University of Naples Federico II (protocol no. 66, approved on 17 January 2019). All participants provided written informed consent before inclusion. The study followed the Strengthening the Reporting of Observational Studies in Epidemiology (STROBE) [27] recommendations and the Initiative on Methods, Measurement, and Pain Assessment in Clinical Trials (IMMPACT) guidelines for chronic pain outcomes [28].

### 2.2. Participants and Eligibility Criteria

Patients with BMS were prospectively enrolled among consecutive subjects referred to the Orofacial Pain Unit of the University of Naples Federico II.

Eligible subjects met the diagnostic criteria for BMS according to the International Classification of Orofacial Pain (ICOP) 2020 criteria [1]. Diagnosis was established after a comprehensive clinical evaluation to exclude local and systemic causes of oral burning and was confirmed by experienced clinicians in oral medicine and orofacial pain.

Patients were excluded if the diagnostic work-up revealed conditions potentially responsible for oral burning or capable of interfering with pain evaluation. Subjects presenting oral mucosal diseases, including candidiasis, oral lichen planus, pemphigus, geographic tongue, or other inflammatory or ulcerative lesions, were not considered eligible. Patients with salivary gland disorders or clinically relevant hyposalivation were also excluded.

Systemic disorders known to be associated with oral burning, such as anemia, hematinic or vitamin deficiencies, Sjögren syndrome, uncontrolled diabetes mellitus, thyroid disease, or other endocrine or metabolic conditions, led to exclusion from the study. Subjects with neurological diseases affecting trigeminal function or with psychiatric disorders requiring recent changes in pharmacological treatment were also excluded.

Patients receiving medications known to induce oral burning, dysgeusia, or xerostomia, as well as those who had undergone recent modifications in systemic or topical therapies that could influence pain perception, were not included. Subjects unable to complete the follow-up evaluation or with incomplete clinical data were excluded.

All included patients received tPGB as part of routine clinical management.

Pregabalin was administered as PregenAQ^®^ 20 mg/mL oral solution (Neuraxpharm, Ascoli Piceno, Italy), a commercially available ready-to-use liquid formulation of pregabalin marketed in Italy. No extemporaneous compounding, dilution, or additional preparation was performed. The solution was supplied in its original bottle with the oral dosing syringe and was stored according to the manufacturer’s recommendations, as no special storage conditions are required. Patients were instructed to apply the prescribed amount directly onto the symptomatic oral mucosa and to avoid eating, drinking, or rinsing immediately after application in order to prolong mucosal contact.

Patients were instructed to swish and spit 7.5 mL of the solution (150 mg PGB) for approximately 5 min, four times daily, without swallowing, to ensure adequate mucosal exposure while minimizing systemic absorption.

No concomitant topical or systemic treatments specifically prescribed for BMS were initiated or modified during the study period. Only patients who completed both baseline (T0) and 3-month follow-up (T1) assessments were included in the final analysis.

### 2.3. Clinical Assessment and Outcome Variables

Clinical and sociodemographic variables were collected at baseline (T0), including sex, age, years of education, family situation, employment status, body mass index (BMI), smoking habits, alcohol consumption, physical activity, systemic comorbidities, current medications, and the Age-Adjusted Charlson Comorbidity Index (AACCI) [29].

All patients underwent a standardized multidimensional clinical assessment aimed at evaluating pain intensity, qualitative pain characteristics, psychological status, sleep quality, and overall clinical severity.

Pain intensity was measured using the Visual Analog Scale (VAS) [30], while qualitative pain perception was assessed using the Short-Form McGill Pain (SF-MPQ) [31]. Psychological status was evaluated using the Hamilton Anxiety Rating Scale (HAM-A) [32] and the Hamilton Depression Rating Scale (HAM-D) [33]. Sleep quality and daytime sleepiness were assessed using the Pittsburgh Sleep Quality Index (PSQI) [34] and the Epworth Sleepiness Scale (ESS) [35]. Global clinical severity was evaluated using the Clinical Global Impression–Severity of illness (CGI-S) [36].

All instruments were administered at baseline and repeated after 3 months (T1) of treatment to evaluate longitudinal changes in pain intensity, psychological burden, sleep quality, and overall clinical severity during topical pregabalin therapy, according to previously published clinical protocols for the multidimensional assessment of chronic oral pain disorders.

### 2.4. Safety and Tolerability

Safety and tolerability were assessed throughout the study. Patients were specifically instructed to report any local or systemic symptoms potentially related to treatment.

At follow-up visits, adverse events were systematically evaluated through structured patient interviews and clinical examination, with particular attention to symptoms relevant to intraoral topical therapy (e.g., mucosal irritation, altered taste, dryness, or discomfort) as well as potential systemic effects such as dizziness or somnolence.

### 2.5. Statistical Analysis

All statistical analyses were performed using Jamovi software (version 2.6.13; The Jamovi Project, 2022).

Given the exploratory nature of the study, no formal sample size calculation was performed. Our decision was justified considering that the enrolled cohort of 100 patients exceeds the median sample size reported in most published clinical trials and observational studies on BMS, supporting the adequacy of the sample for the exploratory purposes of this study [37,38].

Descriptive statistics were used to summarize the study population. Categorical variables were expressed as frequencies and percentages, while continuous variables were reported as median and interquartile range (IQR), given the non-normal distribution of most clinical variables. Normality was assessed using the Shapiro–Wilk test. Changes between baseline (T0) and 3-month follow-up (T1) were analyzed using the Wilcoxon signed-rank test for paired samples. Longitudinal comparisons were performed for all assessed clinical outcomes (VAS, SF-MPQ, HAM-A, HAM-D, PSQI, ESS, CGI-S). To identify predictors of treatment response, univariate and multivariate linear regression analyses were performed using the absolute change in VAS and SF-MPQ score (T1–T0) as the dependent variable, where negative values indicate improvement. In the univariate analyses, each candidate predictor was tested individually. Variables with a *p*-value < 0.20 in the univariate analysis were entered into the multivariable model, following the approach recommended by Hosmer et al. [39]. In addition, HAM-A and HAM-D were included in the multivariable models a priori, given their established clinical relevance in BMS, regardless of their univariate *p*-values. Due to the exploratory nature of the analyses and the number of predictors tested (*n* = 12), Bonferroni correction was applied for multiple comparisons, setting the adjusted significance threshold at *p* < 0.0042 (0.05/12). Percentage changes in VAS and SF-MPQ scores were additionally calculated for responder analyses and graphical representation (waterfall plots). All statistical tests were two-tailed, and statistical significance was set at *p* < 0.05.

## 3. Results

The sociodemographic and clinical characteristics of the study population are reported in Table 1. The sample study consisted of 100 patients diagnosed with BMS, with a clear predominance of females (84%) and a median age of 67 years (IQR 56–72). The median educational level was 12.5 years (IQR 8–13), and the median body mass index was 25.15 (IQR 22.91–28.09). Systemic comorbidities were present in 81% of subjects, with a median Age-Adjusted Charlson Comorbidity Index (AACCI) score of 3 (IQR 1–4). The most frequently reported comorbid conditions included hypercholesterolemia, hypertension, peripheral vascular disease, and endocrine disorders. Concomitant medication use was common, particularly antihypertensive agents, proton pump inhibitors, statins, and antiplatelet drugs.

Clinical features of oral symptoms are reported in Table 2. In addition to burning sensation, xerostomia (67%) and dysgeusia (50%) were the most frequently reported symptoms. The tongue was the most involved site (86%), and symptoms were continuous in 38% of patients, while 46% reported partial improvement during meals.

At baseline (T0), patients presented with moderate-to-severe pain intensity, with a median VAS score of 8 (IQR 7–9) and a median SF-MPQ score of 10 (IQR 7–17). Psychological assessment revealed anxiety (median HAM-A 17, IQR 13–20.25) and depressive symptoms (median HAM-D 15, IQR 11–18). Sleep disturbances were highly prevalent, with a median PSQI score of 7.5 (IQR 6–10), and 91% of patients were classified as poor sleepers. The median CGI-S score was 4 (IQR 3–4), indicating moderate clinical severity of illness. (Table 3).

Table 4 summarizes the longitudinal comparison of clinical outcomes between the baseline (T0) and the three-month follow-up (T1).

Pain intensity (VAS) decreased from a median of 8 (IQR 7–9) at baseline to 5 (IQR 4–6) at follow-up (Wilcoxon W = 3828, *p* < 0.001; effect size = 1.00), while qualitative pain perception (SF-MPQ) decreased from 10 (IQR 7–17) to 6.5 (IQR 4–10; W = 2514, *p* < 0.001; effect size = 0.98). Anxiety symptoms, assessed with the HAM-A, decreased from a median of 17 (IQR 13–20.25) to 15 (IQR 11–18; W = 628, *p* < 0.001), while depressive symptoms, evaluated with the HAM-D, declined from 15 (IQR 11–18) to 12 (IQR 9.75–16; W = 3292, *p* < 0.001). Conversely, PSQI and ESS scores remained relatively stable between T0 and T1 and did not reach statistical significance (PSQI: 7.5 [IQR 6–10] vs. 7.0 [IQR 6–10], *p* = 0.315; ESS: 5.1 [IQR 3–7] vs. 4.5 [IQR 3–7], *p* = 0.624).

A responder analysis was conducted to assess changes in pain intensity based on VAS and SF-MPQ scores. A reduction of at least 20% was observed in 73% of patients. A ≥30% reduction was achieved in 46% of cases, while 16% of patients experienced a ≥50% decrease in pain intensity. A minority of patients (13%) showed no improvement; however, no worsening of pain was observed.

**Figure 1:**
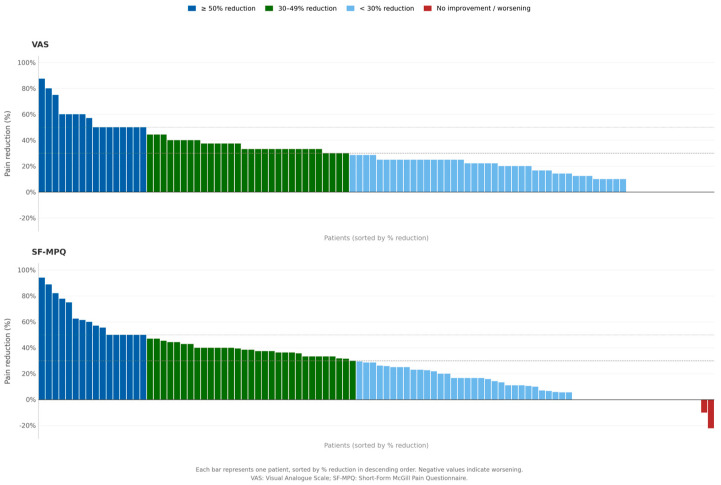
Waterfall Plots of Individual Pain Reduction in BMS Patients Treated with Topical Pregabalin (VAS and SF-MPQ). Each bar represents one patient, sorted in descending order by percentage reduction. Negative values indicate worsening. VAS: Visual Analog Scale; SF-MPQ: Short-Form McGill Pain Questionnaire.

A similar distribution was observed for qualitative pain perception (SF-MPQ), with 61% of patients achieving a ≥20% reduction, 47% reaching the ≥30% threshold, and 16% achieving a ≥50% reduction. However, in contrast to VAS, a subset of patients (21%) showed no improvement or slight worsening, indicating a more heterogeneous response pattern. The distribution of individual responses is visually illustrated in the waterfall plots (Figure 1), where each bar represents a single patient ordered by percentage change. These plots highlight the graded nature of treatment response, the clustering of patients across clinically relevant thresholds, and the greater variability observed in SF-MPQ compared to VAS.

To identify baseline predictors of pain improvement, univariate and multivariate linear regression analyses were performed (Table 5), using the change in VAS score from T0 to T1 (T1–T0) as the dependent variable, where negative values indicate improvement. At the univariable level, baseline VAS score was the only variable significantly associated with pain reduction (β = −0.41; 95% CI −0.508 to −0.192; *p* < 0.001). In the multivariable model, this association remained significant (β = −0.44; 95% CI −0.559 to −0.195; *p* < 0.001), indicating that higher baseline pain levels were associated with greater absolute improvement. Disease duration also reached statistical significance in the multivariable model (β = −0.19; 95% CI −0.021 to −1.39 × 10^−4^; *p* = 0.047). However, given the small magnitude of the regression coefficient and the confidence interval approaching zero, this finding should be interpreted with caution. No other variables were significantly associated with VAS change. The overall model showed acceptable fit (R^2^ = 0.222; adjusted R^2^ = 0.163; *p* = 0.001).

Similar analyses were conducted for qualitative pain perception (SF-MPQ) (Table 6). At the univariable level, both baseline SF-MPQ (β = −0.46; 95% CI −0.330 to −0.145; *p* < 0.001) and disease duration (β = −0.25; 95% CI −0.055 to −0.007; *p* = 0.013) were associated with greater improvement; however, the latter did not remain significant after Bonferroni correction. In the multivariable model, baseline SF-MPQ remained independently associated with pain reduction (β = −0.45; 95% CI −0.326 to −0.138; *p* < 0.001), while disease duration retained borderline significance (β = −0.24; 95% CI −0.052 to −0.007; *p* = 0.009). Given the small magnitude of this effect and confidence intervals approaching zero, this finding should be interpreted with caution. No other predictors were identified. The model showed good fit (R^2^ = 0.28; adjusted R^2^ = 0.25; *p* < 0.001).

No serious adverse events were reported. Two patients (2%) reported a mild and transient unpleasant taste, with no other local or systemic adverse effects observed.

## 4. Discussion

This prospective real-world study provides novel evidence supporting the use of tPGB as a therapeutic option in BMS. Over a 3-month follow-up, treatment was associated with a significant reduction in pain intensity and qualitative pain perception, accompanied by parallel improvements in psychological burden, confirming the multidimensional impact of the intervention.

The present study has several strengths, including the relatively large sample size (n = 100), the prospective design, and the use of a comprehensive multidimensional assessment encompassing pain intensity, qualitative pain perception, and psychological burden.

At the same time, the three-month follow-up period should be interpreted as allowing assessment of short-term clinical response rather than long-term effectiveness. Given the chronic and often fluctuating nature of the condition, longer observation periods may be useful to determine whether the observed improvements are maintained over time and to further characterize the durability of treatment benefit in routine clinical practice.

Within this context, the magnitude of pain reduction observed in our cohort is consistent with previous reports on topical neuromodulators, supporting the clinical relevance of locally targeted therapies in BMS. In our sample, 46% of patients achieved a ≥30% reduction in VAS scores, while 16% reached a ≥50% threshold, indicating a clinically meaningful, although heterogeneous, treatment response. This variability is in line with the well-recognized heterogeneity of BMS, likely reflecting differences in the relative contribution of peripheral and central sensitization mechanisms across patients [40].

Comparable results have been reported with other topical agents. Lebel et al. described a mean reduction of 3.1 ± 2.8 points on a 0–10 scale after topical amitriptyline, with approximately 50% of patients achieving ≥50% pain reduction [20]. Hussein and El-Marssafy further demonstrated a dose-dependent effect, with pain reductions exceeding 60% at higher concentrations [21]. Similarly, Gramacy and Villa reported a median 2-point reduction on a 0–10 NRS with topical gabapentin in a real-world cohort [19]. Topical clonazepam, the most extensively studied agent, has shown comparable efficacy, with randomized data demonstrating a mean reduction of approximately 2.4–2.5 points and responder rates approaching two-thirds of patients [41]. Taken together, these findings suggest that different classes of topical neuromodulators provide consistent, albeit variable, analgesic benefit in BMS.

From a mechanistic perspective, the observed efficacy of tPGB further supports the hypothesis that peripheral nociceptive dysfunction plays a relevant role in BMS pathophysiology [13,42]. Pregabalin binds to the α2δ subunit of voltage-gated calcium channels, reducing calcium influx and inhibiting the release of excitatory neurotransmitters such as glutamate and substance P [43]. This mechanism differs from that of amitriptyline, which primarily blocks voltage-gated sodium channels (Nav1.7, Nav1.8, Nav1.9), suggesting that these agents may target complementary peripheral pathways and providing a rationale for potential combination topical strategies [44].

In addition, experimental evidence supports the biological plausibility and safety of topical pregabalin [45]. Preclinical and translational studies by Xavier et al. have shown that pregabalin-based intraoral formulations are well tolerated and not associated with cytotoxic effects on oral mucosal cells [46,47]. In a clinical setting, these formulations were also associated with a reduction in dentin hypersensitivity without inducing mucosal irritation, supporting the feasibility of local administration and the ability of pregabalin to modulate peripheral nociceptive activity without tissue damage [47].

From a safety perspective, important differences emerge when comparing available topical treatments. Topical amitriptyline has been associated with mild adverse events in approximately 27% of patients, including somnolence and xerostomia, while topical gabapentin has shown adverse drug reactions in around 15–20% of cases, including sedation and oral discomfort [19,20]. Although topical clonazepam is generally well tolerated, mild sedation has also been reported [18]. In contrast, the tolerability profile observed in our cohort appears particularly favorable: only two patients (2%) reported a mild, unpleasant taste, with no other local or systemic adverse events and no worsening of symptoms. This finding is particularly relevant in the BMS population, which is typically older and frequently affected by comorbidities and polypharmacotherapy [15].

The divergent tolerability profiles between gabapentinoids may be partially attributed to their distinct pharmacokinetic behaviors when administered via the oral mucosa. In this study, PGB was delivered as an oral solution (syrup) using a “swish-and-spit” modality, a formulation originally designed for systemic absorption but here repurposed to ensure prolonged mucosal contact.

This delivery method may influence local drug availability while limiting systemic exposure [48]. Both gabapentin and PGB are highly polar, hydrophilic molecules with a negative decadic logarithm of the partition coefficient (logP), which limits their passive transcellular diffusion across biological membranes [23]. Consequently, their absorption depends on carrier-mediated transport, specifically the Large Neutral Amino Acid Transporter 1 (LAT1/SLC7A5) [49]. While LAT1 is highly expressed in the intestinal epithelium and the blood–brain barrier, its representation in the stratified squamous epithelium of the oral mucosa is significantly lower and more variable. In the context of topical administration, the scarcity of LAT1 transporters in the oral epithelium may function as a “pharmacokinetic barrier”, restricting rapid systemic uptake while facilitating drug retention within the mucosal and submucosal layers. Under these conditions, PGB, which exhibits more linear and predictable transport kinetics compared to the saturable absorption profile of gabapentin, can reach effective concentrations at peripheral nociceptive endings. This allows for the targeted modulation of α2δ subunits on voltage-gated calcium channels at the site of origin of pain signaling. This localized mechanism may plausibly contribute to the lower incidence of central nervous system adverse effects, such as sedation and dizziness, typically associated with systemic gabapentinoid therapy. Although these mechanisms require further formal pharmacokinetic validation, they suggest that liquid formulations designed to maximize mucosal contact may allow for a partial dissociation between local analgesic efficacy and systemic toxicity.

This comparison, however, should be interpreted with caution. The adverse-event rates reported for topical amitriptyline, gabapentin, and pregabalin derive from different studies rather than from direct head-to-head comparisons. Therefore, the apparently lower rate observed with topical pregabalin cannot be attributed with certainty to differences in pharmacokinetic properties or LAT1-mediated transport mechanisms. Although this mechanism may represent a plausible hypothesis, alternative explanations, including differences in study design, formulation, concentration, treatment duration, patient characteristics, and adverse-event reporting, may also account for the variability across studies. Direct comparative studies are therefore needed to determine whether topical pregabalin has a genuinely more favorable tolerability profile than other topical neuromodulators. These considerations regarding mucosal pharmacokinetics remain hypothetical, as direct pharmacokinetic data after topical pregabalin application were not collected in the present study. Accordingly, the proposed role of transporter-mediated mucosal handling and local drug availability should be regarded as a plausible mechanistic interpretation rather than a demonstrated finding. Future studies specifically assessing pregabalin concentrations in oral mucosal tissues, saliva, and systemic circulation would help clarify the pharmacokinetic behavior of this approach.

The responder analysis provides additional insight into treatment effects. While 73% of patients achieved at least minimal clinically important improvement (≥20%), only a minority reached the ≥50% threshold. This distribution suggests that tPGB may be particularly effective in a subset of patients, likely those with a predominant peripheral component of pain, whereas individuals with stronger central sensitization may derive more limited benefit. This interpretation is consistent with current models of BMS as a heterogeneous condition involving both peripheral and central mechanisms.

Regression analyses further support this interpretation. Baseline pain intensity emerged as the main predictor of improvement, with higher initial scores associated with greater absolute reductions. Interestingly, longer symptom duration was not significantly associated with pain improvement in the univariate analyses, but emerged as a significant independent predictor in both multivariable models, suggesting that its effect may be partially masked by confounding variables. Given the small magnitude of the regression coefficients and confidence intervals approaching zero, however, this finding should be interpreted with caution.

However, several limitations should be acknowledged. First, the pilot, observational, non-randomized, and uncontrolled design represents the main methodological limitation of the study. The absence of a placebo-controlled group prevents definitive conclusions regarding treatment efficacy or causality and does not allow exclusion of a placebo effect, which is known to be substantial in BMS. In addition, the lack of randomization and blinding may have introduced selection, performance, and assessment biases. The observational design also limits the ability to control for potential confounding factors influencing treatment response, including baseline symptom severity, psychological comorbidities, concomitant medications, and individual variability in disease mechanisms. Second, the lack of a comparative arm precludes direct comparison with other topical or systemic therapies currently used in BMS. Third, the follow-up period was limited to three months, and longer-term data are needed to assess the durability of treatment effects and the persistence of tolerability. Another limitation is that the study did not include a systematic geographic analysis of BMS prevalence or treatment approaches across different world regions. Therefore, the findings should be interpreted in the context of the limited geographical representativeness of the available comparative literature and may not be fully generalizable to populations from underrepresented regions. Furthermore, no a priori sample size calculation was performed, and the sample size was therefore based on the exploratory nature of this preliminary pilot study. This study was not registered in a clinical trial registry; future controlled studies based on these findings will be prospectively registered on an appropriate clinical trial platform. Finally, although the study captures multiple clinical dimensions, it does not allow precise phenotyping of patients according to peripheral versus central mechanisms, which may be critical in predicting treatment response. In addition, the topical use of an oral pregabalin solution represents a non-standardized and off-label approach. However, it should be noted that most topical neuromodulatory therapies currently used in BMS, including clonazepam, amitriptyline, and gabapentin formulations, are similarly administered off-label and lack standardized formulations. Therefore, the present findings should be interpreted cautiously and considered preliminary and hypothesis-generating. They support the rationale for future randomized, placebo-controlled, adequately powered, and prospectively registered studies with longer follow-up and more precise patient phenotyping.

## 5. Conclusions

tPGB may represent a promising and well-tolerated therapeutic option for the management of BMS, with observed improvements in pain intensity and in the broader multidimensional burden of symptoms.

However, BMS remains a complex and heterogeneous condition, in which peripheral nociceptive dysfunction represents only one component of a broader pathophysiological spectrum that may also include central sensitization and psychosocial factors. In this context, the variability in treatment response observed in our cohort suggests that peripheral modulation alone may not be sufficient to address the complexity of symptoms in all patients.

Rather than being viewed as a standalone approach, tPGB may be considered as one potential component of a multimodal therapeutic strategy. Its favorable tolerability and local mechanism of action suggest that it could be explored in selected patients, including in the early phases of treatment, particularly when systemic neuromodulators are not immediately indicated, tolerated, or expected to provide rapid benefit. Topical therapies may also have a potential role in combination with centrally acting agents; however, the possible complementary effects of such approaches remain to be clarified.

Overall, these preliminary findings suggest that topical neuromodulators may have a role in the individualized management of BMS. Nevertheless, given the limitations of the present study, these results should be interpreted with caution. Further randomized controlled studies are needed to confirm these observations, better characterize potential responder profiles, and clarify the optimal positioning of topical pregabalin within multimodal treatment pathways for BMS.

## Figures and Tables

**Table 1:** Sociodemographic profile, risk factors, systemic comorbidities, Age-Adjusted Charlson Comorbidity Index and medications in the 100 BMS patients.

Demographic Variables	BMS Patients
Gender	Frequency (%)
Female Male	84 (84%)
	16 (16%)
Age (in years)	Median [IQR]
	67 (56–72)
Education (in years)	Median [IQR]
	12.50 (8–13)
Family situation	Frequency (%)
Married	75 (75%)
Widowed	15 (15%)
Divorced	6 (6%)
Single	4 (4%)
Employment Status	Frequency (%)
Homemaker	38 (38%)
Retired	30 (30%)
Employed	29 (29%)
Unemployed	3 (3%)
Body Mass Index	Median [IQR]
	25.15 (22.91–28.09)
**Risk factors**	**Frequency (%)**
Smoking	
Never	77 (77%)
<5 cigarettes	3 (3%)
5–10 cigarettes	4 (4%)
10–15 cigarettes	7 (7%)
>15 cigarettes	7 (7%)
e-cig	2 (2%)
Alcohol use	
Never	94 (94%)
Yes (1–2 units/week)	5 (5%)
Yes (>3)	1 (1%)
Physical Activity	
Yes	27 (27%)
No	73 (73%)
**Systemic comorbidities**	**Frequency (%)**
Yes	81 (81%)
No	19 (19%)
Hypercholesterolemia	50 (50%)
Hypertension	48 (48%)
Peripheral vascular disease	18 (18%)
Hypothyroidism	14 (14%)
Gastrointestinal diseases	12 (12%)
Other cardiovascular diseases	11 (11%)
HBV infection	9 (9%)
Atrial fibrillation	5 (5%)
Previous myocardial infarction	5 (5%)
Asthma	4 (4%)
Benign prostatic hypertrophy	4 (4%)
HCV infection	4 (4%)
Neoplastic diseases	3 (3%)
Stroke/TIA	1 (1%)
Hyperthyroidism	0 (0%)
Others	55 (55%)
**Medications**	**Frequency (%)**
Yes	42 (42%)
No	58 (58%)
Beta blockers	17 (17%)
Angiotensin II receptor antagonists (ARBs)	14 (14%)
Proton pump inhibitors	14 (14%)
Statins	12 (12%)
Antiplatelets	11 (11%)
Levothyroxin sodium	8 (8%)
Thiazide diuretics	6 (6%)
Ezetimibe	5 (5%)
Bisphosphonates	4 (4%)
Calcium channel blockers	4 (4%)
ACE-inhibitors	3 (3%)
Steroids	3 (3%)
Blood thinners	1 (1%)
Other	20 (20%)
**AACCI**	**Median [IQR]**
	3 (1–4)

Continuous variables are reported as median (IQR); categorical variables are reported as frequency (%). Data for comorbidities and drug use are reported as the frequency of patients that did have that systemic disorder or used that drug. Abbreviations: 9AACCI, Age-Adjusted Charlson Comorbidity Index; ACE inhibitors, angiotensin-converting enzyme inhibitors; BMI, body mass index; BMS, Burning Mouth Syndrome; HBV, hepatitis B virus; HCV, hepatitis C virus; TIA, transient ischemic attack.

**Table 2:** Prevalence of oral symptoms, location and pattern of symptomatology of the sample of BMS patients.

Oral Symptoms	Frequency (%)
Burning	100 (100%)
Xerostomia	67 (67%)
Dysgeusia	50 (50%)
Globus pharyngeus	35 (35%)
Intraoral foreign body sensation	19 (19%)
Oral dysmorphism	15 (15%)
Sialorrhea	12 (12%)
Tingling sensation	11 (11%)
Itching	7 (7%)
Occlusal dysesthesia	7 (7%)
Dysosmia	4 (4%)
Oral dyskinesia	4 (4%)
Burdening pain	3 (3%)
Allodynia	2 (2%)
Hypoesthesia	2 (2%)
Subjective halitosis	1 (1%)
**Location of Pain/Burning**	**Frequency (%)**
Tongue	86 (86%)
Palate	43 (43%)
Lips	40 (40%)
Buccal mucosa	38 (38%)
Gums	31 (31%)
Floor of the mouth	27 (27%)
Perioral mucosa	25 (25%)
Retromolar trigone	22 (22%)
**Pattern of Symptoms**	**Frequency (%)**
Continuous	77 (77%)
Intermittent	23 (23%)
**Improvement During Meals**	**Frequency (%)**
Yes	46 (46%)
No	54 (54%)

Data for symptoms and location are reported in terms of the frequency of patients who responded “yes”. Abbreviations: BMS: Burning Mouth Syndrome.

**Table 3:** Pain, sleep and clinical evaluation, and psychological profile of the sample of BMS patients at T0.

Pain Evaluation	Median [IQR]
**VAS**	8 (7–9)
**SF-MPQ**	10 (7–17)
**Psychological Profile**	
**HAM-A**	17 (13–20.25)
**HAM-D**	15 (11–18)
**Sleep Evaluation**	**Median [IQR]**
**PSQI total score**	7.5 (6–10)
	**Frequency (%)**
PSQI total score < 5	9 (9%)
PSQI total score ≥ 5	91 (91%)
	**Median [IQR]**
**ESS total score**	5.07 (3–7)
**Clinical Global Impression Severity of Illness**	**Median [IQR]**
**CGI-S**	4 (3–4)

Continuous variables are reported as median (IQR); categorical variables are reported as frequency (%). Abbreviations: BMS: Burning Mouth syndrome; CGI-S: Clinical Global Impressions Severity of Illness; ESS: Epworth Sleepiness Scale; HAM-A: Hamilton Anxiety Rating Scale. HAM-D: Hamilton Depression Rating Scale; PSQI: Pittsburgh Sleep Quality Index; SF-MPQ: Short-form McGill Pain Questionnaire; VAS: Visual Analog Scale.

**Table 4:** Pain evaluation, anxiety, and depression symptoms were compared at the first visit and after three months.

Variable	T0	T1			
	**Median [IQR]**		**Wilcoxon W**	***p***	**Effect Size**
**VAS**	8 (7–9)	5 (4–6)	3828	**<0.001**	1
**SF-MPQ**	10 (7–17)	6.5 (4–10)	2514	**<0.001**	0.98
**HAM-A**	17 (13–20.25)	15 (11–18)	2628	**<0.001**	0.91
**HAM-D**	15(11–18)	12(9.75–16)	3292	**<0.001**	0.95
**PSQI**	7.5 (6–10)	7.0 (6–10)	1842	0.315	0.11
**ESS**	5.1 (3–7)	4.5(3–7)	1935	0.624	0.05
**CGI-S**	4 (3–4)	3(1–4)	3150	**<0.001**	0.68

Values are reported as median (IQR). Comparisons between T0 and T1 were performed using the Wilcoxon signed-rank test. Effect sizes are reported for within-subject changes. Abbreviations: HAM-A: Hamilton Anxiety Rating Scale, HAM-D: Hamilton Depression Rating Scale; SF-MPQ: Total Pain Rating Index; VAS: Visual Analog Scale.

**Table 5:** Univariate and multivariate linear regression analysis of predictors of pain reduction (VAS) in BMS patients.

	Univariate Analysis				Multivariate Analysis			
		95% CI				95% CI		
Predictor	β	Lower	Upper	*p* Value	β	Lower	Upper	*p* Value
**Age (years)**	−0.14	−0.041	0.001	0.165	−0.12	−0.037	0.008	0.192
**Gender: Male vs. Female**	−0.25	−1.25	0.453	0.355	—	—	—	—
**Disease Onset (months)**	−0.14	−0.018	0.004	0.181	−0.19	−0.021	−1.39e^−4^	**0.047**
**Smoking: Yes vs. No**	0.13	−0.537	0.951	0.582	—	—	—	—
**Alcohol Use: Yes vs. No**	0.19	−1.01	1.63	0.643	—	—	—	—
**HAM-A (T0)**	−0.15	−0.086	0.011	0.130	−0.14	−0.107	0.037	0.335
**HAM-D (T0)**	−0.11	−0.075	0.021	0.27	0.13	−0.038	0.099	0.380
**VAS (T0)**	−0.41	−0.508	−0.192	**<0.001**	−0.44	−0.559	−0.195	**<0.001**
**SF-MPQ (T0)**	−0.13	−0.077	0.016	0.199	0.02	−0.04	0.052	0.825
**PSQI (T0)**	0.07	−0.064	0.138	0.474	—	—	—	—
**ESS (T0)**	−0.03	−0.114	0.082	0.752	—	—	—	—
**CGI (T0)**	−0.17	−0.498	0.031	0.083	0.02	−0.258	0.305	0.868

Outcome variable: Change in VAS score from T0 to T1 (negative values indicate improvement). Univariate analysis: Each predictor was tested individually. Multivariate analysis: Model includes predictors selected based on univariable screening (*p* < 0.20). Depression was included anyway because of its established role in BMS patients. Model fit: R^2^ = 0.222, Adjusted R^2^ = 0.163, F (7,92) = 3.76 (*p* = 0.001). Abbreviations: HAM-A: Hamilton Anxiety Rating Scale; HAM-D: Hamilton Depression Rating Scale; SF-MPQ: Total Pain Rating Index; VAS: Visual Analog Scale; β: regression coefficient; CI: Confidence Interval; ESS: Epworth Sleepiness Scale; PSQI: Pittsburgh Sleep Quality Index.

**Table 6:** Univariate and multivariate linear regression analysis of predictors of pain reduction (SF-MPQ) in BMS patients.

	Univariate Analysis				Multivariate Analysis			
		95% CI				95% CI		
Predictor	β	Lower	Upper	*p* Value	β	Lower	Upper	*p* Value
**Age (years)**	0.005	−0.052	0.055	0.960	—	—	—	—
**Gender: Male vs. Female**	−0.32	−3	0.772	0.244	—	—	—	—
**Disease Onset (months)**	−0.25	−0.055	−0.007	0.013 *	−0.24	−0.052	−0.007	**0.009**
**Smoking: Yes vs. No**	0.13	−0.603	2.68	0.213	—	—	—	—
**Alcohol Use: Yes vs. No**	0.09	−1.65	4.19	0.392	—	—	—	—
**HAM-A (T0)**	−0.07	−0.145	0.073	0.514	−0.13	−0.220	0.079	0.351
**HAM-D (T0)**	0.04	−0.086	0.128	0.700	0.20	−0.035	0.249	0.137
**VAS (T0)**	0.01	−0.373	0.395	0.954	—	—	—	—
**SF-MPQ (T0)**	−0.46	−0.330	−0.145	**<0.001**	−0.45	−0.326	−0.138	**<0.001**
**PSQI (T0)**	0.09	−0.123	0.325	0.375	—	—	—	—
**ESS (T0)**	0.04	−0.177	0.258	0.713	—	—	—	—
**CGI (T0)**	−0.003	−0.603	0.589	0.98	—	—	—	—

* Considering Bonferroni correction for multiple comparisons, the critical value for *p* should be 0.05/12 = 0.0042. Outcome variable: Change in SF-MPQ score from T0 to T1 (negative values indicate improvement). Univariate analysis: Each predictor was tested individually. Multivariate analysis: Model includes predictors selected based on univariable screening (*p* < 0.20). Depression and anxiety were included anyway because of their established role in BMS patients. Model fit: R^2^ = 0.28, Adjusted R^2^ = 0.25, F (4,95) = 9.24 (*p* < 0.001). **Abbreviations:** HAM-A: Hamilton Anxiety Rating Scale; HAM-D: Hamilton Depression Rating Scale; SF-MPQ: Total Pain Rating Index; VAS: Visual Analog Scale; β: regression coefficient; CI: Confidence Interval; ESS: Epworth Sleepiness Scale; PSQI: Pittsburgh Sleep Quality Index.

## Data Availability

Data are available upon request to the corresponding author.

## Abbreviations

AACCI	Age-Adjusted Charlson Comorbidity Index
ACE inhibitors	Angiotensin-Converting Enzyme inhibitors
ARBs	Angiotensin II Receptor Blockers
BMI	Body Mass Index
BMS	Burning Mouth Syndrome
CGI-S	Clinical Global Impression–Severity
CI	Confidence Interval
ESS	Epworth Sleepiness Scale
HAM-A	Hamilton Anxiety Rating Scale
HAM-D	Hamilton Depression Rating Scale
HBV	Hepatitis B Virus
HCV	Hepatitis C Virus
ICOP	International Classification of Orofacial Pain
IMMPACT	Initiative on Methods, Measurement, and Pain Assessment in Clinical Trials
IQR	Interquartile Range
LAT1	Large Neutral Amino Acid Transporter 1
MPQ	McGill Pain Questionnaire
NRS	Numeric Rating Scale
PGB	Pregabalin
PSQI	Pittsburgh Sleep Quality Index
SF-MPQ	Short-Form McGill Pain Questionnaire
STROBE	Strengthening the Reporting of Observational Studies in Epidemiology
tPGB	Topical Pregabalin
TIA	Transient Ischemic Attack
VAS	Visual Analog Scale
